# Public preferences for reducing health inequality in the US: a national survey

**DOI:** 10.1186/s12910-026-01405-7

**Published:** 2026-02-13

**Authors:** Kyoko Shimamoto, Tim Doran, Richard Cookson

**Affiliations:** 1 Keio University, Tokyo, Japan; 2 Department of Health Sciences, University of York, York, UK; 3 Centre for Health Economics, University of York, York, UK

**Keywords:** Health equity, Health inequality, Health inequality aversion, Health related social welfare function, Equity-efficiency trade-off, US

## Abstract

**Background:**

There are sometimes trade-offs between improving total health and reducing health inequality among social groups. When faced with such trade-offs, decision makers need information about public values. However, although evidence of this kind has been collected in a limited number of countries, there is little evidence of this kind available for the United States.

**Methods:**

We used a national online survey of the US general population (aged 18–69) and applied a modified version of a standard benefit trade-off questionnaire used in several other countries, involving pairwise policy choices with different levels of health benefit and inequality. Proportions in qualitative health inequality categories were calculated, along with a simple index based on healthy years foregone and health inequality aversion parameters based on Atkinson and Kolm indices. Multivariable regression analyses were conducted to explore within-country heterogeneity by age, gender, income, education and geographic region.

**Results:**

Around two thirds (65.9%) of respondents in the analytic sample (*n* = 440) had positive health inequality aversion, of which 20.7% were classified as “Weighted Prioritarian”, 4.3% as “Maximin” and 40.9% as “Strict Egalitarian”. The remaining respondents were mostly “Pro-Rich” (32.7%) rather than “Health Maximisers” (1.4%). The median respondent was “Weighted Prioritarian” and weighed health gains to the poorest fifth of people six to seven times more highly than health gains to the richest fifth (Atkinson 9.1; Kolm 0.1). Health inequality aversion was lower in the Midwest region of the US compared to the Northeast region, but otherwise was not closely correlated with observed respondent characteristics.

**Conclusions:**

Median views about health inequality aversion in the US were similar to those in Japan and the UK, but there was a greater degree of polarisation with higher proportions of respondents at both extremes of the distribution (“Pro-Rich” and “Strict Egalitarian”).

**Supplementary Information:**

The online version contains supplementary material available at 10.1186/s12910-026-01405-7.

## Background

Health inequalities between more and less socially advantaged groups have been receiving increasing attention globally [1]. The US is an especially interesting country to study in this respect, since health inequality gaps have been widening in the US since around 1980 [2, 3]. This increase started earlier and has been larger and more sustained than in the UK, where health inequality only started increasing since 2010 [4, 5] and in other high income countries health inequality has not been increasing in recent decades – such as Canada [6] and most European countries [7].

The causes of health inequality are many and complex, including wider social determinants of health and inequalities in access to health care and related financial protections [8, 9]. The Covid-19 pandemic shone a spotlight on inequalities on this kind in the US and globally [10] and highlighted difficult dilemmas for policy-makers, who may be forced to make trade-offs between reducing health inequality and improving average health – the so-called equity-efficiency trade-off.

To make informed decisions about such trade-offs, policy makers need to understand public attitudes towards health inequality. Researchers have attempted to quantify public perceptions using social choice experiments to elicit ‘health inequality aversion’ parameters which represent the degree of concern for reducing health inequality versus improving total health. The theoretical basis for this approach was originally developed in the field of welfare economics, in the form of a simple “social welfare function” (SWF) that articulates trade-offs between total wellbeing and its distribution using an “inequality aversion” parameter [11]. Building on this standard, health economists have employed an analogous health-related social welfare function (HRSWF) with a “health inequality aversion” parameter, for example, an Atkinson or Kolm parameter which is based on simple single parameter level dependent Social Welfare Functions (SWFs) [12–15]. Health inequality aversion can be estimated in a range of ways [16–19], and other questionnaire methods and SWF frameworks are available, as discussed later in the Discussion section. However, the most common method is the benefit trade-off (BTO) approach as already used in several other countries and allows comparisons with others [12, 14].

To date, studies have mainly focused on European populations but social choice experiments have also been applied in Asia–Pacific settings, for example Australia and Japan [20, 21]. There is some evidence that US populations would support sacrificing efficiency (total population health gain) to improve equity in health outcomes to some extent, but to date this evidence is limited [18]. A most recent US study employed an adapted BTO, yet applied an intensive screening approach, finding that the majority was health inequality averse and the median was prioritarian [22]. Recent findings from the UK and Japan using the same methods without such screening suggested there are similarities in the extent of health inequality aversion in different populations [23]. However, given variations in socio-demographics, social inequalities, health distributions and cultural attitudes, it is likely that patterns of health inequality aversion will differ in important ways between countries.

The US health care system is primarily privately provided with targeted public programs covering those over the age of 65 (Medicare), with low incomes (Medicaid) or affiliated with the military (The Veterans Health Administration). The greater emphasis on private provision, coupled with the higher national levels of economic inequality [24], suggests that the US population may be less inequality averse on average than the populations of the UK and Japan. The US also has a higher level of political polarization, which effects attitudes towards public health [25], hence there may be correspondingly greater polarization with respect to health inequality aversion. Despite growing interest in equity-informative health economic evaluations, such as Distributional Cost Effectiveness Analysis (DCEA) [26], the approach has yet to be extensively applied [21, 27, 28], largely due to data constraints, including lack of information on health inequality aversion parameters. Improving understanding of the range of views would assist decision makers in developing policies that align with public sentiment.

In this study, we aim to gauge public views on health inequality aversion in the US, and to compare these to views previously elicited for UK and Japanese populations. The preference pattern for health inequality aversion is assessed using a benefit trade-off approach used in a UK study [14], with minor modifications for the US, so that we can compare the US findings with findings elsewhere. Categorical response patterns are analysed and health-related social welfare functions (HRSWFs), in the form of Atkinson and Kolm indices, are estimated to quantify equity weightings across countries; and in-country differences are assessed by socio-demographics in the US, compared to the UK and Japan where the health system and insurance are driven primarily by the government and farer than the US’s market-based system. It is hypothesized that, in consideration of the US health system, median health inequality aversion is lower in the US, compared to the UK and Japan; that variability in health inequality aversion is higher in the US, compared to the UK and Japan; and that health inequality aversion varies between socio-demographic groups within the US.

## Methods

### Study setting and data collection

This study was conducted March 2023. Data were collected at the national level, from the general population aged 18–69. Participants were recruited from a registered survey panel by an online survey company Cint and their partnering companies in the US involving the survey panels of multiple consortiums and covering such academic research activities. Quota sampling based on the national population statistics was used to approximate the distribution of the national population [29]. These data included age (using ten-year groups), gender (including male, female and other), geographic locations (using a four regional boundary classification based on the national census), education (using five main categories) and income (using quintiles) – as outlined in detail in the below section on “measures”. This data collection approach is consistent with the comparative Japanese study [23], while the UK study employed a population weighting approach using the national statistics [14].

The analytic sample was selected using the same response classification system as previous UK and Japanese studies [14], which classifies responses as “consistent” or “inconsistent” using the fifteen pre-defined categories for the policy choice response (see Table 1). This categorisation is explained in the proceeding section of “Measures” as adopted after the UK methods [14]. The survey company employed a five-percentile cutoff to exclude participants with the shortest answer time from the sample, resulting in the inclusion of the respondents who took at least four minutes of answer time. The final survey sample comprised 965 participants, which is a similar study sample size compared to the previous studies in the UK and Japan, and the valid analytic sample was 440 participants that provided “consistent” responses as per the pre-defined response classification system (equivalent to 45.6% of the survey sample). The distribution of the socio-demographic characteristics of both the final full survey sample and the valid analytic sample is reported later under “Results”.

**Table 1:** Definitions and implied parameter values for each response category for the US study

**Rank**	**Category**	**Response pattern**	**Health gain for Poor, in Program B**	**Total health sacrificed**	**Health inequality aversion**		**Implied weight for the poorest group**	
					**Atkinson, e**	**Kolm, a**	**Atkinson, e**	**Kolm, a**
1	Pro-rich 1	AAAAAAA	8.5	−1.5	−1.69	−0.02	0.7	0.7
2	Pro-rich 2	= AAAAAA	8	−1	−1.16	−0.02	0.8	0.8
3	Pro-rich 3	BAAAAAA	7.5	−0.5	−0.60	−0.01	0.9	0.9
4	Health Maximiser	B = AAAAA	7	0	0.00	0.00	1.0	1.0
5	Weighted Prioritarian 1	BBAAAAA	6.5	0.5	0.65	0.01	1.2	1.1
6	Weighted Prioritarian 2	BB = AAAA	6	1	1.38	0.02	1.4	1.3
7	Weighted Prioritarian 3	BBBAAAA	5.5	1.5	2.21	0.03	1.6	1.6
8	Weighted Prioritarian 4	BBB = AAA	5	2	3.20	0.05	2.0	1.9
9	Weighted Prioritarian 5	BBBBAAA	4.5	2.5	4.45	0.06	2.6	2.5
10	Weighted Prioritarian 6	BBBB = AA	4	3	6.19	0.09	3.8	3.5
11	Weighted Prioritarian 7	BBBBBAA	3.5	3.5	9.13	0.13	7.2	6.4
12	Maximin	BBBBB = A	3	4	Infinity	Infinity	Infinity	Infinity
13	Egalitarian 1	BBBBBBA	2.5	4.5	NA	NA	NA	NA
14	Egalitarian 2	BBBBBB =	2	5	NA	NA	NA	NA
15	Egalitarian 3	BBBBBBB	1.5	5.5	NA	NA	NA	NA

This categorisation system is adopted from the UK study [14] as reported in “Appendix B: Response Categorisation”

An extended 26 category system is used for sensitivity analysis built on the original fifteen response categories, with additional eleven response patterns that included an “equally good” answer option in two or three consecutive pairs. All respondents are still classified within one of the original 15 main categories, and the additional eleven categories are sub-categories that allow additional responses to be classified as “consistent” and counted within one of the 15 main categories. The additional categories are as follows: Pro-rich 3 [= = AAAAA], Health Maximiser [= = = AAAA], Weighted Prioritarian 1 [B = = AAAA], Weighted Prioritarian 2 [B = = = AAA], Weighted Prioritarian 3 [BB = = AAA], Weighted Prioritarian 4 [BB = = = AA], Weighted Prioritarian 5 [BBB = = AA], Weighted Prioritarian 6 [BBB = = = A], Weighted Prioritarian 7 [BBBB = = A], Maximin [BBBB = = =], and Egalitarian 1[BBBBB = =]

### Questionnaires

The questionnaire for this research study was developed and published as the British English language instrument for eliciting health inequality aversion by Robson et al. [14] (See Appendix in the supplemental materials). The original questionnaire was edited in American English by all members of the author team who have some familiarity with US English, with support from a US resident. This questionnaire was also adapted for a Japanese study [23]. For this study questionnaire, baseline health was assumed as 58 healthy life years among the poorest fifth and 72 years among the richest fifth, with a 14-year gap. These figures had to be guesstimated for the purpose of this study, based on published estimates of adult life expectancy by income group in the US and of the social distribution of Quality Adjusted Life Expectancy (QALE) in the UK [3, 30, 31]. The comparative studies and their baseline health status data from the UK (with a 12-year gap) and Japan (with a 10-year gap) were considered for comparability, and we guesstimated that the US gap would be 2 years larger than the UK gap. Guestimation was needed because at the time the survey was designed and piloted there was no directly relevant estimate of the social distribution of QALE at birth in the US. Such data have subsequently become available only after the time of this survey [21, 27], though focusing on geographic social vulnerability and race/ethnicity sub-groups rather than income groups.

The main component of the questionnaire was policy choice questions about equity-efficiency trade-offs in terms of gains in healthy life years (i.e., years in full health, years in the state of perfect health) among the richest and poorest fifths in the US context. Respondents were asked to choose between two government programmes, ‘A’ and ‘B’, with different gains of healthy life years for the richest and poorest groups, resulting in the varied gap in healthy life years among the two groups. In each scenario, Programme A provided greater gains for the richest group consistently (i.e., seven years for the richest and three years for the poorest throughout the seven scenarios); and Programme B mostly greater gains for the poorest group (i.e., eight years in the first scenario and two years in the seventh scenario for the poorest, vis-à-vis three years consistently for the richest). Respondents had to choose the most preferred option: “Program A”, “Program B”, or “Program A and B are equally good”. For example, in the first scenario, Programme A increased healthy life years by seven years for the richest and by three for the poorest, and Programme B provided gains of three and eight years respectively. In each proceeding scenario, increases for the poorest group under Programme B were reduced, but all other gains remained constant.

In addition, other questionnaire components included the following: i) an introductory video with a duration of three minutes (See Appendix) viewed after the first round of policy choice question and before the second-round choice, as translated from the Japanese video that was adapted as a shortened summary version from the original UK video to improve understanding of the questionnaire [32] in a more efficient manner; ii) priming questions translated from the Japanese questionnaire adapted from the UK questionnaire, which introduced the concept of health and income inequality prior to the main policy choice question and asked about the participant’s perceptions, views and opinions on these inequalities and policy priorities [14, 23]. The participants completed seven pairwise policy choices, both before and after viewing the introductory video, in the same way as the original UK study (See Table S1 in the supplementary file). We used the responses obtained after the video as our primary analysis, but also compared how far responses shift after viewing the video.

### Measures

#### Categorisation of the policy choice

**Fig. 1:**
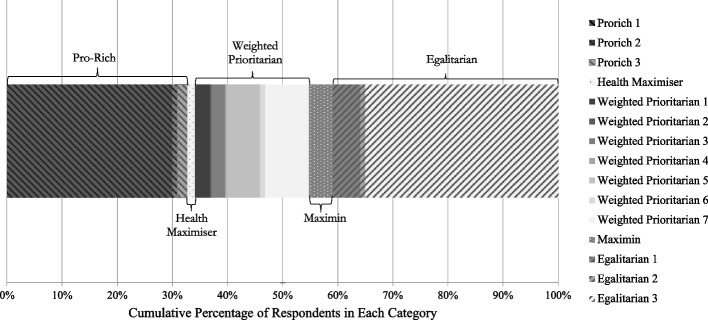
Categorical responses (valid analytic sample *n* = 440, US). Frequencies for each category are as follows: Prorich 1 = 132 observations, Prorich 2 = 4 observations, Prorich 3 = 8 observations, Health Maximiser = 6 observations, Weighted Prioritarian 1 = 12 observations, Weighted Prioritarian 2 = 1 observations, Weighted Prioritarian 3 = 11 observations, Weighted Prioritarian 4 = 1, Weighted Prioritarian 5 = 27 observations, Weighted Prioritarian 6 = 4 observations, Weighted Prioritarian 7 = 35 observations, Maximin = 19 observation, Egalitarian 1 = 22 observations, Egalitarian 2 = 4 observations, and Egalitarian 3 = 154 observations

Responses to the policy choice question were categorised into five major response groups and fifteen categories per the original UK study (Table 1 and Fig. 1). The major groups comprise: “Pro-Rich” – prefer health gains to the better-off; “Health Maximiser” – concerned only with increasing total health; “Weighted Prioritarians” – give greater weight to the health of the worse-off; “Maximim” – concerned only with improving the health of the worst-off; and “Strict Egalitarian” – willing to sacrifice potential health benefits to the worst-off to reduce inequality [14]. The classification was based on the point at which respondents “switch” from preferring Programme B to preferring Programme A in the scenario, or become indifferent between the two programmes, which was also used to elicit the health inequality aversion parameter.

#### The Health-Related Social Welfare Function (HRSWF)

Health-related social welfare functions (HRSWFs) are used to quantify health inequality aversion using the following two parameters. The Atkinson index is concerned with relative inequality, and the Kolm index is concerned with absolute inequality in lifetime health [33].

These concepts of HRSWF are built on the traditional concepts of welfare economics, in particular the concept of Equally Distributed Equivalent (EDE) level of income. The EDE income is defined as the mean level “which if equally distributed would give the same level of social welfare as the present distribution” [11]. This concept is transformed into the EDE level of health, which provides an index of social welfare standardized to the mean level of health [14]. Equations for the EDEs used in this analysis are:

1 $$EDE_{Atkinson} = \bar{H} . \left[ \sum\nolimits_{i} \left( \frac{H_{i}}{\bar{H}} \right)^{1-\varepsilon} f(x_{i}) \right]^{1/(1-\varepsilon)} $$

2 $$EDE_{Kolm} = \bar{H} - \left[ \left(\frac{1}{a}\right) log \sum e^{a.(\bar{H} - H_{i})} f(x_{i})\right]$$

The inequality aversion parameters in these equations are *ε (epsilon)* and *α (alpha)* for the Atkinson and Kolm indices respectively, where both are unbounded. The greater the inequality aversion parameter values (i.e., the value of *ε* epsilon and *α* alpha), the greater the aversion to inequality. *H*_*i*_ is the level of health (healthy life years) for sub-group *i*; $\overline{H }$ is the mean level of health for the entire population; and *f(x*_*i*_*)* is the proportion of the population in the subgroup *i*. This analysis allows the elicitation of negative parameters for respondents reporting “inequality seeking” ethical judgement, as well as positive parameters reporting “inequality averse” judgement per the UK study [14].

#### Socio-demographic variables

The following key socio-demographic characteristics of respondents were recorded and employed for quota sampling methods: age, gender, education, income and geographic region. These demographic variables and recoding categories are the same as the preceding Japanese study [23] for consistency and comparison. Age was assessed as both a continuous and categorical variable: teenagers aged 18–19, adults aged 20–29, aged 30–39, aged 40–49, aged 50–59, and aged 60–69 (reference group: aged 60–69). Gender was categorized as male, female, and other for those responded as “other” or “do not answer” (reference group: male). Education was categorized as “high school or less” or “higher education” (i.e., technical college, 2-year college education or higher) (reference: high school or less), based on the initial major five category option (i.e., elementary school, high-school, lower college, university, graduate school level, or other). Household income was measured in quintiles, in reference to the recent national household annual income data (lowest 20% quintile – USD 28,007 or less; lower 20–40% quantile – from USD 28,008 to USD 55,000; middle 40–60% quintile – from USD 55,001 to 89,744; higher 60–80% quintile – from USD 89,745 to USD 149,131; and highest 20% quintile – above USD 149,132 or more) (reference: the lowest 20% income quintile). Geographic regions were classified into four regions, using the census regional boundary: Northeast (New England and Middle Atlantic divisions), Midwest (East North Central and West North Central divisions), South (South Atlantic, East South Central, and West South Central divisions), and West regions (Mountain and Pacific divisions) (reference: Northeast) [29].

### Analysis

Analysis comprised five major steps: i) descriptive analysis of response patterns and the socio-demographic characteristics of the US respondents, using the pre-defined fifteen category system, based on which the analytic samples were classified within one of the categories; ii) comparative analysis of the major response patterns in the US, in comparison to the UK and Japan using descriptive and bivariate analysis; iii) calculation of HRSWFs for each respondent using Excel for each pre-defined classification category based on the equations under “Measure (for Atkinson and Kolm indices)” as adopted from the UK study [14] and the US study population (i.e., a median for the study population average); iv) multivariable linear regression analysis (OLS) of respondents’ levels of health inequality aversion and socio-demographic characteristics; and v) sensitivity analysis of health inequality aversion using different cut-off points of analytic sample definitions (e.g., an extended 26 category system, and questionnaire answer time ranges to complete the whole questionnaire). An extended twenty-six category system is built on the original fifteen response categories, with additional eleven response patterns that included an “equally good” answer option in two or three consecutive pairs (See Table 1). All respondents are still classified within one of the original fifteen main categories, and the additional eleven categories are sub-categories that allow additional responses to be classified as “consistent” and counted within one of the fifteen main categories.

Health inequality aversion was estimated based on the point at which respondents switched preferences or became indifferent between the two programmes, per the response of each sample at the second-round policy choice after viewing the video. This point represented health gains to the poorest group under Programme B, ranging from 1.5 years (Egalitarian 3) to 8.5 years (Pro-Rich 1). This was rescaled to create a simple index of health inequality aversion based on healthy years by subtracting the initial value from seven, resulting in the range from – 1.5 years (Pro-Rich 1) to + 5.5 years (Egalitarian 3), with 0 indicating a Health Maximiser. This index allows a simple linear regression approach to investigating associations between health inequality aversion and socio-demographic variables, which is easier to interpret than ordered logistic regression with fifteen response categories. A 0.5 change in healthy years along this index is equivalent to a one rank change in response category. In the regression analysis, standard errors were adjusted for clustering by geographic region based on the census boundary, being consistent with the comparative Japanese study by the authors that employed a few combined regions.

All analyses were conducted using Excel and STATA version 17. Ethical approval was obtained from the Research Ethics Committee of the School of Health Management, Keio University, in February 2021.

## Results

### Descriptive analysis of the US respondents

#### Socio-demographic characteristics

Descriptive analysis results of the respondents’ demographic characteristics are shown in Table 2. Following quota sampling, the survey sample generally approximated the distribution of the national population in terms of most of the selected demographic characteristics. The distribution of the analytic sample (*n* = 440) was similar to the survey population, suggesting the approximation of the national-level demographic pattern of the analytic sample, with an exception of education with which the survey and analytic sample comprised a higher proportion of higher education group compared to the national statistics.

**Table 2:** Socio-demographic characteristics of the survey participants (US full survey sample = 965; valid analytic sample = 440)

Socio-demographics	Full survey sample		Valid analytic sample		National stats
	Frequency	Proportion (%)	Frequency	Proportion (%)	Proportion (%)
Age					
18–19	90	9.3	45	10.2	9
20–29	178	18.5	90	20.5	19
30–39	187	19.4	88	20.0	19
40–49	169	17.5	69	15.7	18
50–59	178	18.5	74	16.8	18
60–69	163	16.9	74	16.8	17
Gender					
Female	483	50.1	213	48.4	50
Male	479	49.6	226	51.4	50
Other	3	0.3	1	0.2	Not available
Geographic region					
Northeast	172	17.8	74	16.8	17
Midwest	207	21.5	98	22.3	21
South	356	36.9	158	35.9	39
West	230	23.8	110	25.0	23
Education					
Highschool or less	298	30.9	99	22.5	38
College or more	667	69.1	341	77.5	62
Household income quintiles					
Highest 20%	209	21.7	121	27.5	20
Higher 20–40%	145	15.0	67	15.2	20
Middle 40–60%	158	16.4	73	16.6	20
Lower 60–80%	211	21.9	93	21.1	20
Lowest 20%	242	25.1	86	19.6	20

Gender categories included, male, female, other, or do not answer. In the table, “other” comprises the respondent who selected “other” and “do not answer”

US census data were referenced for quota sampling; the census sampling framework was not the same as this study sample framework (e.g., age distributions), thus the national statistics distribution was adjusted to resonate with this study sample framework, as reported under the national stats column and proportions

#### Categorical responses to the policy choice question

**Fig. 2:**
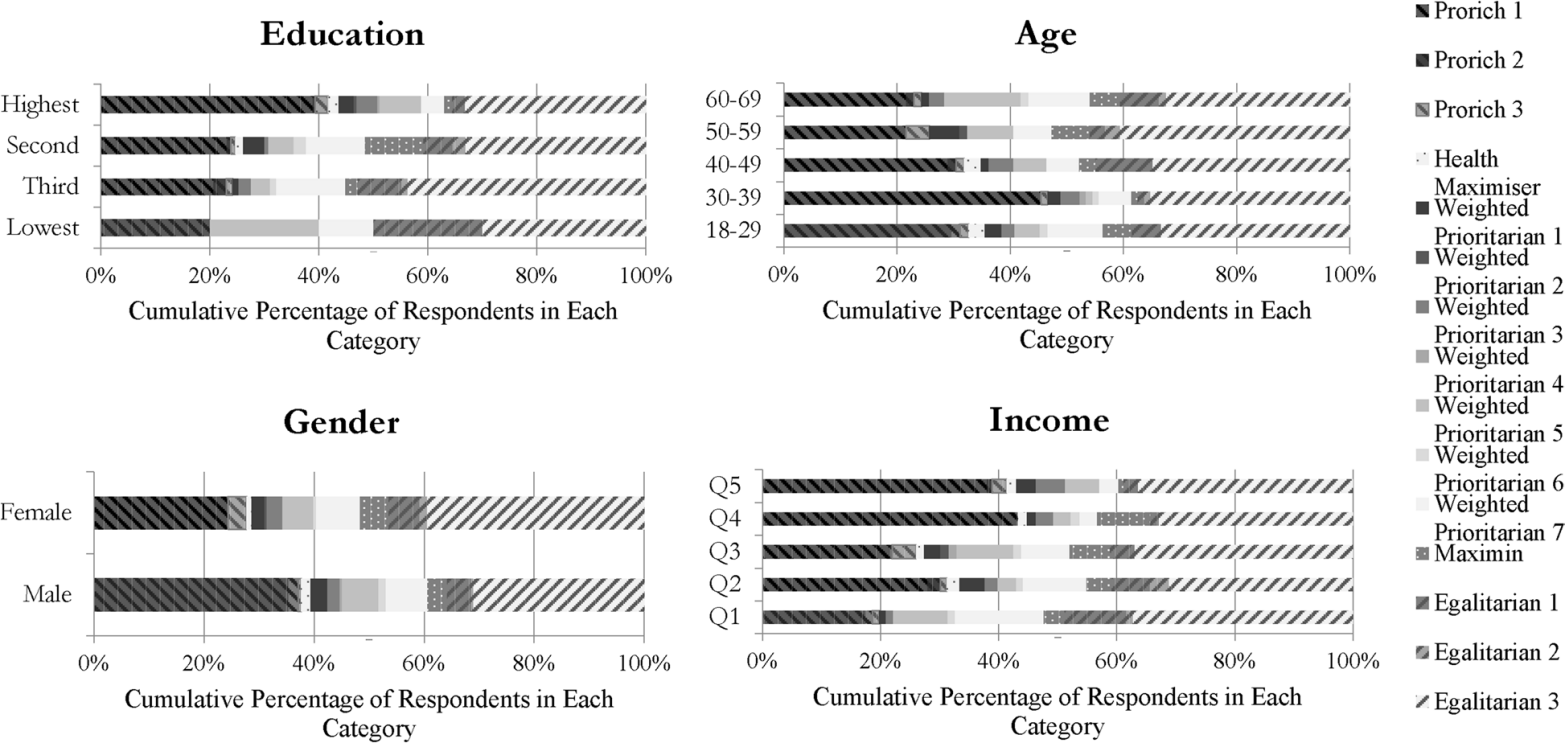
Sub-group analysis of the categorical responses by age, gender, education and income (valid analytic sample *n* = 440, US). Note: Education is categorized as follows: Highest = University and higher; Second = Lower college level; Third = high school level; Lowest = primary and junior high school. Other (*n* = 2 in the analytic sample) is not shown in the figure. Gender is categorized as male, female and other/do not answer. The last category (*n* = 1) is not shown in the figure. Income is categorized as household income quintiles from Q1 = the poorest fifth (lowest 20%) to Q5 = the richest fifth (highest 20%)

The distribution of responses to the policy choice question is shown in Table 3 and Fig. 1. The majority of the responses were classified either as “Pro-rich (32.7%)” or “Strict Egalitarian (40.9%)”, whilst the remainder mostly comprised “Weighted Prioritarian (20.7%)”. Around the two-thirds of the respondents (65.9%) were willing to trade-off total health in order to reduce health inequality to some extent; almost the half (45.2%) had extremely high health inequality aversion being either “Maximin” or “Strict Egalitarian”; and only 1.4% were strict “Health Maximisers”. The results of sub-group analysis by demographic characteristics are shown in Fig. 2 and Figure S1 (in the supplementary file), suggesting variations by sub-group in terms of age, gender, education, income and geographic region respectively. These associations were examined in subsequent regression analysis.

**Table 3:** Descriptive statistics of the five major response categories (valid analytic sample *n* = 440)

Major response category	Frequency	Proportion (%)
Pro-rich	144	32.7
Health Maximiser	6	1.4
Weighted Prioritarian	91	20.7
Maximin	19	4.3
Egalitarian	180	40.9

“Weighted Prioritarian”, “Maximin” and “Egalitarian” categories all exhibit some degree of health inequality aversion, in the sense that they are willing to trade-off some total health for reducing health inequality (in total, 65.9%); the “Maximin” and “Egalitarian” exhibit extremely high health inequality aversion (in total, 45.2%)

### Comparative analysis of the US, the UK and Japanese studies

**Fig. 3:**
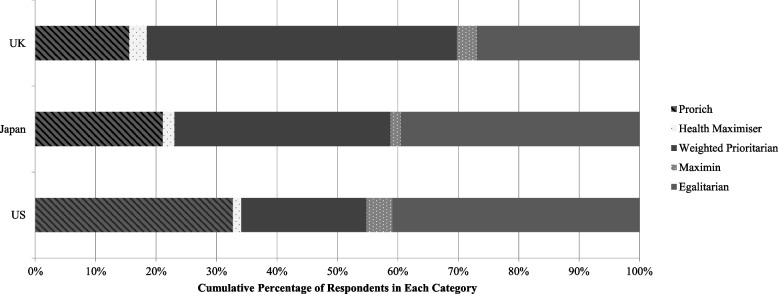
International comparison of the five main categorical responses (valid analytic samples for UK *n* = 244; Japan *n* = 473; US *n* = 440). Note: Detailed descriptive statistics are reported in each referenced study. Reference: (1) UK: Robson, M., Asaria, M., Cookson, R., Tsuchiya, A., & Ali, S. (2017). Eliciting the level of health inequality aversion in England. *Health economics, 26*(10), 1328–1334. https://doi.org/10.1002/hec.3430. (2) Japan: Shimamoto, K., Doran, T., & Cookson, R. (2025). Public preferences for reducing health inequality in Japan: A national survey. *Value in Health Regional Issues*, 101111. https://doi.org/10.1016/j.vhri.2025.101111. (3) US: Shimamoto, K., Doran, T., & Cookson, R. (2025). Public preferences for reducing health inequality in the US: A national survey. (This study)

Figure 3 shows the descriptive comparison of the US with the UK and Japan using the major five categories – Pro-rich, Health Maximiser, Weighted Prioritarian, Maximin and Strict Egalitarian. The median category was the same across the three countries, Weighted Prioritarian; however, the degree of polarisation in terms of having large proportions of both “Pro-Rich” and “Strict Egalitarian” differed substantially across the three countries. In the US there were 32.7% Pro-rich and 40.9% Strict Egalitarian, whereas in the UK the corresponding proportions were 15.6% and 27.0%, respectively, and in Japan 21.1% and 39.5%. Association tests using chi-square test suggest that the US is significantly different from the UK and Japan, with a lower proportion of health inequality averse respondents in the US, whilst there is no statistically significant difference (*p* < 0.05) between the UK and Japan.

### Health inequality aversion parameters – Atkinson and Kolm indices

Table 4 shows the elicited health inequality aversion parameter values and corresponding EDE level of health from the US study, including the specific parameter values of Atkinson and Kolm as well as implied equity weights for the poorest group compared with the richest. We found a median health inequality aversion parameter value of 9.1 for Atkinson and 0.1 for Kolm in this US study populations, based on the HRSWF estimation for each respondent. Based on this, and the baseline levels of health, we calculated the implied equity weights representing the marginal rate of substitution of health between the poorest fifth of the population and the richest fifth. At initial levels of healthy life years, incremental health gains to the poorest fifth of people in society were weighted approximately six to seven times as highly as incremental gains to the richest fifth (Atkinson implied weight to the poorest group 7.2; Kolm implied weight 6.4).

**Table 4:** Health inequality aversion parameter values (valid analytic sample *n* = 440)

HRSWF	Median (95% Confidence Intervals)	Implied weight for the worst-off fifth compared with 1 for the best-off fifth
Atkinson	9.1 (4.5–9.7)	7.2
Kolm	0.1 (0.1–0.2)	6.4

### Multivariable regression analysis

Table 5 shows the results of multivariable linear regression analyses (OLS), as well as the unadjusted OLS associations with no control variables. In the model, the dependent variable is the simple index of health inequality aversion, based on the health gains to the poorest fifth group in Programme B. This value is connected with each response category of the fifteen-category system, whereby each 0.5 increase in healthy years in terms of the simple index represents a one rank increase in response category, and positive values indicate that the individual is health inequality averse to some extent.

**Table 5:** Associations between simple health inequality aversion score in years and respondent characteristics, with and without adjustment for other characteristics (valid analytic sample *n* = 440)

		**Dependent variable:** **Simple health inequality aversion score in years**					
		**[Model 1] Adjusted model**			**[Model 2] Unadjusted model**		
		**Coefficient**	**95% Confidence Interval**		**Coefficient**	**95% Confidence Interval**	
Independent variables							
Female		0.658	- 0.941	2.257	0.771	0.216	1.326
Others	(Ref. = Male)	1.245	- 0.429	2.918	1.085	- 4.790	6.961
Age 18–19		0.148	- 0.573	0.869	0.835	- 0.085	1.755
Age 20–29		- 0.530	- 2.088	1.029	- 0.601	-1.293	0.090
Age 30–39		- 0.783	- 2.483	0.917	- 0.834	- 1.529	- 0.139
Age 40–49		- 0.219	- 1.313	0.874	0.038	- 0.731	0.808
Age 50–59	(Ref. = Age 60–69)	0.031	- 1.093	1.155	0.571	-0.175	1.317
Higher education	(Ref. = High school or less)	- 0.543	- 1.697	0.611	- 0.941	-1.605	- 0.277
Second lowest income quintile		- 0.682	- 1.460	0.095	0.023	-0.662	0.709
Middle income quintile		- 0.200	- 1.247	0.847	0.420	-0.331	1.171
Second highest income quintile		- 0.916	- 2.744	0.913	-0.659	- 1.436	0.117
Highest income quintile	(Ref. = Lowest income quintile)	- 0.660	- 1.914	0.594	-0.706	- 1.330	- 0.083
Midwest region		- 0.230	- 0.426	- 0.035	-0.208	- 0.881	0.464
South region		0.046	- 0.109	0.201	0.070	- 0.513	0.653
West region	(Ref. = Northeast)	0.003	- 0.277	0.283	-0.035	- 0.681	0.611
Intercept		3.097	1.313	4.881	NA		
Model statistics							
R-squared		0.062			NA		
Root MSE		2.940			NA		

The presented model employs OLS, using the simple health inequality aversion score in years from −1.5 (Pro Rich 1) to + 5.5 (Egalitarian 3), with 0 indicating a health maximiser. It is calculated as 7 minus the trade-off point in years. Higher values indicate greater aversion to health inequality, and positive values indicate that the individual is health inequality averse

Standard errors are adjusted for four clusters per the geographic region variable, and robust coefficients are reported from multivariable regression analysis. Model 1 is the adjusted model, which is multivariable OLS controlling for other respondent characteristics. Model 2 is the unadjusted model, which is a simple OLS examining the bivariate association between the concerned independent variable and the dependent variable (thus the intercept and model statistics vary for each characteristic and are not reported)

Region of residence is significantly associated with health inequality aversion. For example, compared to Northeast region respondents, Midwest region respondents are slightly less health inequality averse, by—0.23 years in terms of the simple index (i.e. just under half a rank shift in the fifteen response categories). Other regions, namely South and West regions, do not show statistically significant differences from the Northeast. Differences across income quintile groups showed borderline statistical significance only (*p* < 0.10), with lower health inequality aversion in the second lowest income quintile than the lowest quintile by—0.68 years in terms of the simple index (i.e., more than one rank shift in the fifteen response categories). There was no statistically significant association of health inequality aversion with age, gender or education, after controlling for these selected demographic characteristics of the respondents.

### Sensitivity analysis

Three kinds of sensitivity analysis were conducted, in precisely the same way as the two previous UK and Japan studies. The first sensitivity analysis divided the analytic sample according to the time taken for the completion of the whole questionnaire. Respondents were grouped based on additional time taken for questionnaire completion in excess of the video viewing duration of three minutes as follows: i) five minutes or more (*n* = 394); ii) ten minutes or more (*n* = 217); and iii) fifteen minutes or more (*n* = 104). The population average of health inequality aversion parameters for these three groups was slightly different from each other – Weighted Prioritarian 7 for group 1; Weighted Prioritarian 6 for group 2; and Weighted Prioritarian 5 for group 3.

The second sensitivity analysis excluded fewer “invalid” responses by allowing some patterns of response that are nearly consistent with our theoretical framework. To do this we allowed responses where “equally good” was given as the response in two or three consecutive pairs (See Table 1). For these eleven additional patterns, the second “equally good” was assumed to be the indifference point among the three points. This broader inclusion criterion resulted in an additional thirty-two respondents being classified as valid and consequently a larger analytic sample (n = 472), yielding the analytic sample proportion of 48.9%. The population average of health inequality aversion parameter (i.e., a median) was the same as the main analysis, i.e., Weighted Prioritarian 7.

The third sensitivity analysis included responses that were likely to suggest a degree of instability, whereby two B-to-A switching points were observed – as a possible one-off error that is subsequently corrected (e.g., BBBABAA). In this case, we randomised which switching point to accept as the correct one, and additional five responses were included. The median though did not change after the main analysis.

## Discussion

### Statement of principal findings

On average, around two thirds of the respondents (65.9%) to our US survey had positive health inequality aversion, when using our analytical sample (*n* = 440). However, there was considerable polarisation of views. The majority of responses were classified either as “Pro-rich” (32.7%) or “Strict Egalitarian” (40.9%), whilst the remainder mostly comprised “Weighted Prioritarian” (20.7%) – a median category. In terms of health inequality aversion parameters, the median respondent weighed health gains to the poorest fifth of people approximately six to seven times more highly than health gains to the richest fifth (Atkinson 9.1; Kolm 0.1). Health inequality aversion was lower in the Midwest region of the US compared to the Northeast region, but otherwise was not closely correlated with observed characteristics.

In the US, a lower proportion of respondents were health inequality averse (65.9%), compared to the UK (81.5%) and Japan (79.6%). There was also a greater degree of polarisation in response patterns, with the majority of responses being in one of the two extreme categories of “Pro-rich” or “Strict Egalitarian” (32.7% and 40.9% respectively). This pattern is notably distinct from the UK, where the majority were Weighted Prioritarian (51.0%) with only 15.6% being Pro-rich and 27.0% Strict Egalitarian. Similar to the US, Strict Egalitarian was the most common category (39.5%) in Japan, followed by Weighted Prioritarian (35.7%) and then Pro-rich (21.1%).

Finally, there was little evidence of variation in views by income in the US but some evidence of differences in health inequality aversion by geographic region. Using the census regional boundaries comprising the four regions, Midwest region had less health inequality aversion compared to Northeast region, whilst there was no statistically significant difference in the comparison of Northeast region with South or West region. In Japan, regional differences were also distinct, besides significant income group differences suggesting self-interest, i.e., lower income respondents were more health inequality averse. This was not a part of the main analysis in the UK.

### Strength and weakness of the study

This study was one of the first to quantify public preferences for reducing health inequality in the US. We used a standard questionnaire that has been extensively applied in different countries, making minor modifications to ensure consistency across settings, enabling cross-country comparison. As anticipated, our results suggest that attitudes towards equity-efficiency trade-offs in US populations differ from UK and Japanese populations, with lower overall levels of health inequality aversion.

New methods in this field are emerging [34] but have not yet been extensively tested or used for comparison between different countries. We therefore used the standard method which – as reported in previous studies [12–14] – has some limitations. First, as with all approaches, there are likely to be framing effects of unknown magnitude relating to the presentation of experimental scenarios, although all comparisons were positive (relative gains in health) [35, 36]. Relatedly, respondents may tend to be categorical in their thinking [37] and relatively insensitive to both baseline health and quantitative magnitudes of health inequalities resulting from trade-offs [38, 39]. This may potentially generate a bias whereby changing the baseline health inequality gap may change the attitudes and responses of respondents in unknown directions and magnitudes and consequently, the estimated health inequality aversion parameter values.

Second, aversion to health inequality among social advantage groups is generally higher than aversion to ‘pure’ health inequality or variation between individuals/groups whose income or social status is unknown, and it is not known how far this reflects bias or genuine concern for the “double disadvantage” of groups experiencing both social disadvantage and health disadvantage, i.e., asymmetry associated with inter-relationship between health and income [34]. Economic inequality has been recognized as a key driver of health inequality in the US [2, 3, 8], as well as racial disparities [40], so further investigation of health inequality aversion among economic groups and its interaction with other social advantage groups (e.g., by race/ethnicity) is warranted.

Third, the analytic sample comprises approximately half of the survey sample (45.6% for the base analysis, and 48.9% for the sensitivity analysis), due to most responses not being consistent with the pre-defined response category system. The “inconsistent” responses comprise those who switched multiple times between Program A and B, choose an indifference (i.e., “A and B are equally good”) for more than three times, and/or selected unexpected opposite directions of switch from Program A to B at least once. This high rate of “inconsistent” responses is similar to the original study in the UK, and is likely due to the unfamiliar and cognitively demanding nature of the equity-efficiency trade-off task that respondents are being asked to wrestle with. This is a common limitation of the benefit trade-off studies, and there are serious challenges in this field of research. The online survey is particularly at risk of these issues, which is prone to less serious engagement than the in-person interview, although there are emerging new approaches using an online survey, e.g., estimation of pooled and participant-specific social preferences while accounting for noise [34].

Fourth, we used an online self-reported questionnaire, which may have produced less considered responses, although excessively quick responses were excluded from the analysis per the standard data screening procedure of the survey company. There is nevertheless a risk that the observed polarisation may be partly due to some respondents giving the quickest and easiest possible set of answers – i.e. always choosing A or B – rather than taking the time and effort to think carefully about these cognitively demanding trade-off questions and give responses that reflect the full nuances of their considered opinions. The uncertain level of understanding among the US samples might have influenced on such response patterns, which is unknown due to the lack of individual interview procedures in this particular US study and should be addressed in future studies to ensure the full understanding of the tasks, ideally using a mixed-method.

Fifth, observed variables were limited in this study, as it was not possible to collect some variables of interest due to the regulation of the contracted survey company, such as race/ethnicity and political ideology. These sociodemographic variables, as well as other related variables (e.g., health insurance status) could potentially be explored in future studies and used to investigate within-country heterogeneities. Sixth, the relatively small survey sample size (n = 965) may not have been nationally representative, although descriptive statistics for the sample approximate national statistics, with the exception of educational level which was relatively high in the survey sample. Seventh, our estimates of Quality Adjusted Life Expectancy (QALE) by income quintile group are based on approximations as no direct data were available apart from related estimates, such as life expectancy [3, 21, 27, 30, 31]. Eighth, framing effects, ordering of policy options and priming questions may have caused biased some responses [41, 42]. Ninth, detailed methodologies for this field of research, including establishing study power and testing for validity and reliability, are still under development. Finally, sociocultural differences might have affected responses, although the influence of linguistic differences will be marginal between American and British English.

### Meaning of the study: explanations and implications

The greater polarization of views about health inequality aversion in the US compared to the UK and Japan may reflect the greater degree of political polarization in the US with respect to issues relating to social and economic inequality [43–45]. Polarization amplifies ideological divisions and increases the likelihood that attitudes towards public health interventions will be based on political affiliation rather than personal circumstances or moral considerations. For example, self-identified Democrats are almost twice as likely as self-identified Republicans to agree that there is too much economic inequality in the country [46], and increasing political polarization plays a significant role in explaining divergence in Americans' attitudes toward equitable health and health care [47]. In the US, Democrat-aligned voters are more likely to support social safety nets and a role for government in ensuring access to health care and reducing inequalities. Republican-aligned voters are more likely to view universal health care as an overreach of government power and a threat to personal freedoms, free-market principles and individual responsibility [48]. Political polarization on this issue would have implications for the kinds of policies administrations at the state and federal levels would be likely to support. As we could not measure political affiliation, further investigations are warranted to examine the association between such polarization and preferences for reducing health inequality in the US. This study generally agrees with the recently published US study using extensive screening, which showed the same median of prioritarian yet no such polarisation [22]. This difference may suggest that there might be greater misunderstanding or more confused responders in this study without screening, and/or genuinely greater polarisation in the US. Future comparative studies could further investigate such screening effects.

We found evidence of geographical heterogeneity, which is consistent with existing evidence from global systematic reviews on the elicitation of equity-efficiency tradeoffs [16]. There is evidence that “regional segregation” in health is increasing in the US [2], which may generate greater regional differences in health inequality aversion. Such geographic differences have complex historical, sociodemographic and cultural origins, which could be explored through additional studies with greater breadth and granularity, covering a greater range of contributory variables.

Equity-informative health economic evaluation, such as Distributional Cost-Effectiveness Analysis (DCEA), requires a health inequality aversion parameter [26]. Our international comparisons provide median estimates ranging from 9.1 for the US to 13.9 for Japan, which could potentially be used as benchmarks for equity-informative economic evaluations in other high-income settings. However, this study demonstrates that country-specific health inequality aversion parameters, accounting for within-country variation, should ideally be estimated, given that potential cultural differences were suggested that may possibly explain observed and unobserved variations.

### Conclusions

In conclusion, this is one of the first studies to examine public preferences for reducing health inequality in the US using an international standard questionnaire allowing cross-country comparisons. The average level of health inequality aversion in the US appears to be similar but slightly lower than in the UK and Japan, with greater polarisation and more respondents adopting extreme pro-rich or pro-poor positions. Our findings imply both between and within country heterogeneities, and more studies are needed to refine measurement methods and further quantify health inequality aversion parameters across a range of settings. These findings will need to be integrated into public health policy and service development decisions to ensure that public preferences on the trade-offs between efficiency and equity inform the delivery of public health and related services.

## Supplementary Information

Supplementary Material 1.

## Data Availability

Data access will be considered if approved by the research ethics committee of Keio University.
