# Supplementary material for: Public preferences for reducing health inequality in the US: a national survey
Source: BMC Med Ethics. 2026 Feb 13;27:129. doi: 10.1186/s12910-026-01405-7 (PMC13359883; doi:10.1186/s12910-026-01405-7)
Supplement: Supplementary file 1 — Supplementary Material 1. [file 12910_2026_1405_MOESM1_ESM.pdf]

Appendices

Table S1: Distribution of responses by the five major categories (five-by-five table of pre-video and post-video responses. USA analytic n=235)

	**Second round choice**					***Total***
**First round choice**	Pro-rich	Health maximiser	Weighted prioritarian	Maximin	Egalitarian	
Pro-rich	55	0	3	0	11	*69*
Health maximiser	0	2	1	0	0	*3*
Weighted prioritarian	10	0	43	1	3	*57*
Maximin	0	0	3	6	3	*12*
Egalitarian	5	0	11	4	74	*94*
Total	*70*	*2*	*61*	*11*	*91*	*235*

Note:
This table includes analytic samples that provided a "consistent (or valid)" response for the both 1st and 2nd round choices. This analytic sample is reduced from the final analytic sample (n=440) that provided a "consistent" response for the 2nd round choice, because observations were excluded from this five-by-five table that provided "consistent" response for the 2nd round choice but an "invalid" response for the 1st round choice that were 205 observations.

Figure S1: Descriptive statistics of response categories by geographic region (USA analytic n=440)

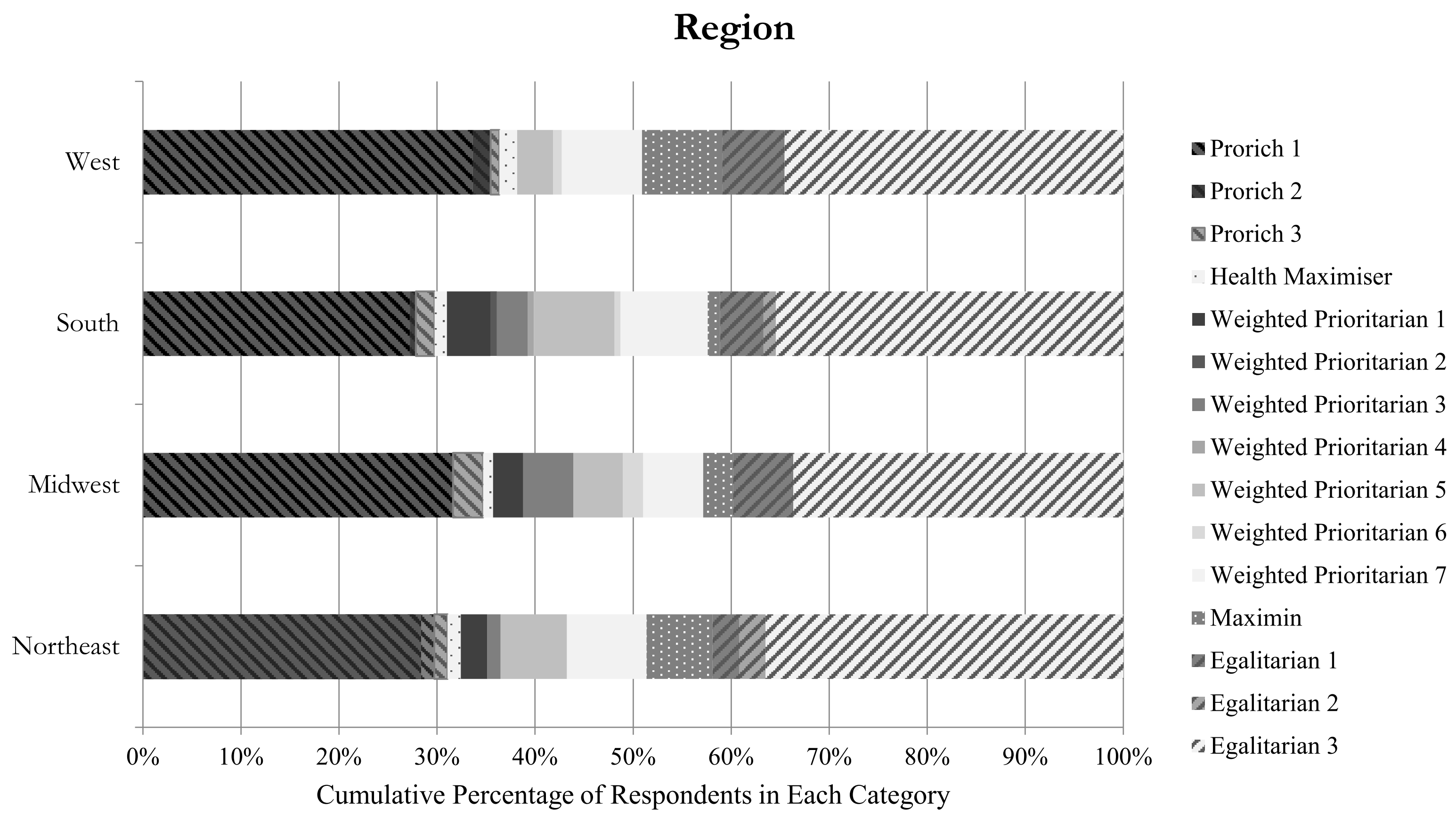

Figure S2: Online trade-off questions for the USA study

TRADE OFF QUESTION

Imagine that you are asked to choose between two large government programs which will improve population health. Both programs cost exactly the same.

Who benefits?

Program	Population Group	Before	Change	After
Program A	Richest Fifth	72	+7	79
	Poorest Fifth	58	+3	61
Program B	Richest Fifth	72	+3	75
	Poorest Fifth	58	+8	66

These are gains in years of life in full health over the average person's lifetime.

When making a decision, it is important to remember the following:

- We cannot pay for both programs — a choice must be made
- "Equally good" means you don't mind which one is chosen
- Both programs cost exactly the same
- The only difference between the programs is the gain to the poorest and richest fifth
- The middle three fifths of the population are not affected

Which program should the government choose?

1.

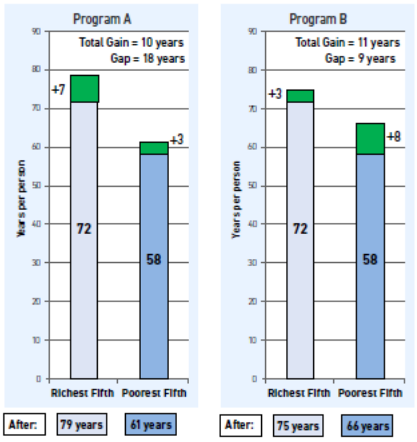

Program A

Program A and B are equally good

Program B

Now imagine it is more difficult than we thought to benefit the poorest fifth. For each of the following comparisons please choose one.

2.

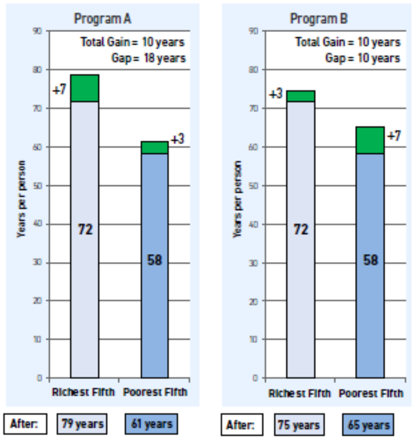

Program A

Program A and B are equally good

Program B

3.

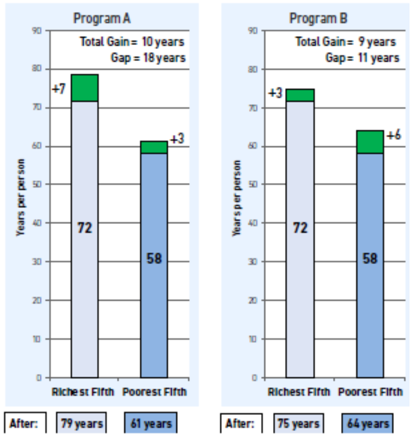

Program A

Program A and B are equally good

Program B

4.

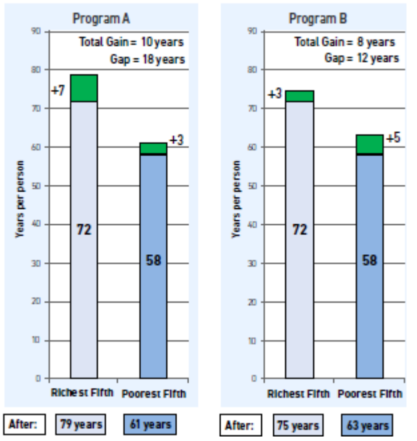

Program A

Program A and B are equally good

Program B

5.

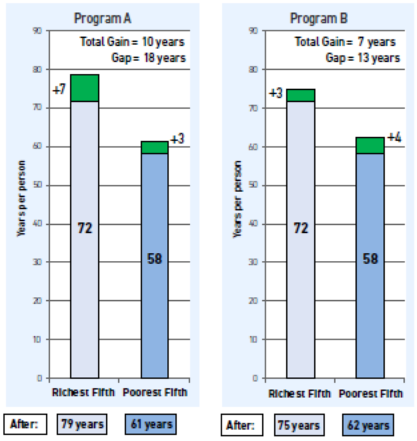

Program A

Program A and B are equally good

Program B

6.

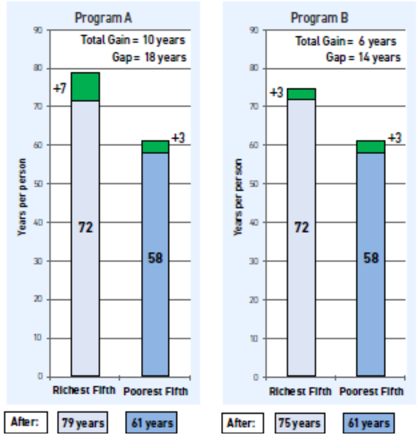

Program A

Program A and B are equally good

Program B

7.

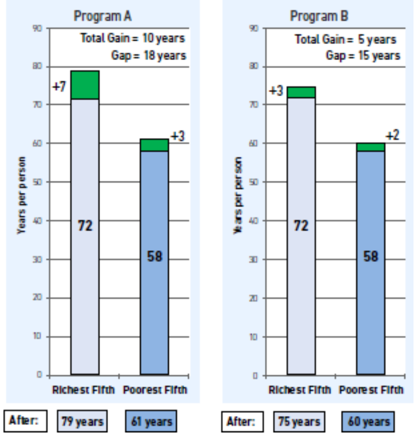

Program A

Program A and B are equally good

Program B

Note: The original full English version of the questionnaire for the UK study is available on the website of the University of York: https://www.york.ac.uk/che/research/equity/economic_evaluation/publicviews/

Appendix: Health inequality aversion introductory video transcript for the USA study

Narrator: Now that you've seen our question about health disparity trade-offs, how will you respond? It's a tricky question to answer, because it raises an ethical dilemma. In this short animation, we have four characters, who will explain their positions to you.

Eddie the Equalizer: Hi! I'm Eddie the Equalizer. Health disparity is unjust. I find it shocking that in the US and other parts of the world, rich people live much longer and healthier lives than poor people. We should do everything we can to reduce these unjust disparities in health. That's why I will always choose Program B—because it gives more healthy years to the poor than the rich and so reduces health disparity.

Rational Rita: Hi! I'm Rational Rita. I have a strong view about which options you should definitely NOT choose. We shouldn't choose a "levelling down" option that gives worse health to the rich and the same health to the poor when we compare Program B to Program A. Rather than "levelling down", I would choose Program A.

Harry the Health Maximizer: Hi! I'm Harry the health maximizer. What matters is improving people's health. Everyone's health matters equally—improving rich people's health is no more and no less important than improving poor people's health. I will almost always choose Program A—because Program A almost always delivers more total health than Program B.

Balanced Bambra: Hi! I'm Balanced Bambra. They all make good points. But they all take things to extremes. I suggest a sensible, pragmatic solution. I will choose Program B to start with, to reduce health disparity. But then at some point, I will switch to Program A when the total health gain from Program B becomes too small. And I will switch BEFORE Program B becomes a "levelling down" option. I want to take a balanced compromise approach.

Narrator: Do you agree with Eddie the Equalizer, who would always choose Program B because it reduces health disparity? Or Rational Rita, who would never choose Program B when it's a "levelling down" option. Or Harry the Health Maximizer, who would almost always choose Program A because it gives the greatest total improvement in population health? Or finally, do you agree with Balanced Bambra, who will start by choosing Program B and then switch at some point to Program A. The choice is yours!

Note: The text was shortened from the original UK version. This short version was used for the Japanese study.
